# Utility of point of care viscoelastic haemostatic assays for trauma patients in the emergency department

**DOI:** 10.1186/s13049-025-01388-1

**Published:** 2025-04-24

**Authors:** Andrew Richard Coggins, Vinh Dat David Nguyen, Leonardo Pasalic, Murari Ramesh, Kush Wangoo

**Affiliations:** 1 Department of Emergency Medicine, Westmead Hospital, Hawkesbury Road, Sydney, NSW 2145 Australia; 2 Western Sydney Local Health DistrictWestmead Hospital, Hawkesbury Road, Sydney, NSW 2145 Australia; 3 Department of Haematology, Westmead Hospital, Hawkesbury Road, Sydney, NSW 2145 Australia

**Keywords:** Blood bank & transfusion medicine, Trauma management, Accident & emergency medicine

## Abstract

**Background and objectives:**

Traumatic haemorrhage often requires initiation of a massive haemorrhage protocol (MHP). The primary aim of this exploratory Emergency Department (ED) study was to examine the utility of point of care Viscoelastic Haemostatic Assays (VHA) in terms of accuracy. The primary outcome was prediction of the need for massive transfusion (MT) at 24-hours.

**Methods:**

Prospective observational study of consecutive trauma patients investigated with reported using STARD guidelines. Patients in an Australian ED setting < 1-hour from triage enrolled in a three-year window. The point-of-care device used was a TEG6s™ (Haemonetics, Braintree, MA, USA). The primary outcome was accuracy VHA testing for predicting MT delivery at 24-hours (an internationally recognised of massive transfusion was used). Other trauma outcomes such as product transfusion, injury severity score (ISS) and demographics were recorded. For analysis of accuracy the cohort was divided into *VHA-normal* (*n* = 44) and *VHA-abnormal* (*n* = 70) binary groups. Secondary outcomes included utility of TEG6s™ individual components and accuracy of VHA when combined with validated MHP decision scores.

**Results:**

Among eligible cases (*n* = 114) in-patient mortality was 7.0% with 91.2% receiving transfusion. Presence of (any) abnormal VHA result provided a 73.6% (95%CI 59.7–84.7) sensitivity and 49.3% (95%CI 36.1–62.3) specificity for predicting MT. Citrated Functional Fibrinogen (CFF) component had a higher performance for MT *“rule-in”* specificity (86.9%). When VHA was combined with validated MHP decision scores performance was increased. For example, normal VHA with Trauma Associated Severe Haemorrhage score < 8.5 was observed to yield a sensitivity of 96.2% for MT requirement rule-out. Further studies should examine if VHA test parameters can be added or (replace INR) in the existing clinical scores used to make decisions about transfusion in ED patients.

**Conclusion:**

The standalone performance of early VHA testing in the ED setting was insufficient to reliably for predict a need for massive transfusion.

**Supplementary Information:**

The online version contains supplementary material available at 10.1186/s13049-025-01388-1.

## Highlights

Viscoelastic haemostatic assays (VHA) such as Rotational Thromboelastometry (ROTEM) and Thromboelastography (TEG) are now available in Emergency Department (ED) settings. Whist utility in the operating room is well-established, VHA has an uncertain role as a point of care test in the ED.

This study adds to an emerging body of work examining novel and point of care tests in this area. Among trauma patients that received VHA we observed a sensitivity of 72.1% and specificity of 60.4% for prediction of MT within 24-hours.

The addition of the VHA result to existing MHP decision rules was noted to increase accuracy in predicting an endpoint of MT at 24-hours. A ‘rule out’ result for both the decision rule(s) and VHA test resulted in a high sensitivity for predicting a need for MT within 24-hours.

**Supplementary Information:**

The online version contains supplementary material available at 10.1186/s13049-025-01388-1.

## Introduction

Traumatic shock remains a leading cause of preventable death with haemorrhage often a diagnostic and management challenge in the Emergency Department (ED) [1–3]. Consideration of the need for activating a Massive Haemorrhage Protocol (MHP) is common in this setting. Administration of blood components in trauma may be associated with trauma-induced coagulopathy (TIC) and cause significant iatrogenic problems [3–7]. An important clinical question in the ED for trauma teams is early identification of who will need MHP. Identification of patients who are at increased risk of MHP is important to ensure blood products are available for such cases. However, accurate prediction of product use can be challenging using existing clinical decision scores [7]. 

Any test(s) that predict massive transfusion (MT) requirements contribute provide useful information to treating teams [7–9]. Of note, conventional coagulation tests are currently included in the existing validated clinical decision scores (i.e., Vandromme and Schreiber scores) [6–8]. For example, international normalised ratio (INR), which is commonly cited as as a valid trigger for MHP, is often slow to be processed. INR results are not typically available to treating trauma teams when deciding on MHP initiation in the ED. On the other hand, VHA tests such as thromboelastography (TEG) and rotational thromboelastometry (ROTEM) have the potential to provide useful real-time data that support decisions [5, 10, 11, 12, 13]. As the result of such uncertainly about the utility of such tests we planned this prospective study. The goal was to add to contribute to the current conversation in the literature regarding how to use VHAs in real-world practice [14, 15].

There is some evidence to support the adoption of VHA in an ED setting [5, 16]. Observational studies of VHA in trauma concluded that VHA may predict the need for transfusion but the ITACTIC study indicated that using a VHA-guided strategy did not result in a mortality benefit [5, 17]. An accompanying editorial noted that VHA assisted in the early recognition of TIC and was beneficial especially in head-injured patients [5, 18]. A 2015 paper supported wider use and concluded that VHA is a “*valid marker for TIC and predictor for massive transfusion*”. These authors reported “*moderate sensitivity*” for predicting coagulopathy (73%) and need for a MT (77%) [8].

To summarise, VHA has been used outside of ED for some time but its role at the point of care in ED remains unclear [3, 8, 19]. Our hypothesis was that the use VHA at the point of care (< 1 h from admission) could be helpful in predicting MT requirements. Additionally, we set out to also examine whether the early VHA results could be combined with existing validated trauma MHP prediction scores and therefore act as an adjunct decision-making in combination with other tools such as the Vandromme score.

## Materials and methods

### Study setting and design

A prospective observational study of consecutive ED patients investigated with VHA < 1-hour from arrival. The study utilised a registry of VHA results (2018–2021) and electronic medical record (EMR) data. Reporting of the study followed the standards for Reporting of Diagnostic Accuracy Studies (STARD) statements.

#### Ethical approval

Obtained from the Western Sydney Local Health District committee (*Ref/PID2207*). The study was characterised as low-negligible risk with waiver of individual consent approved.

### Eligibility, glossary and definitions

**Fig. 1:**
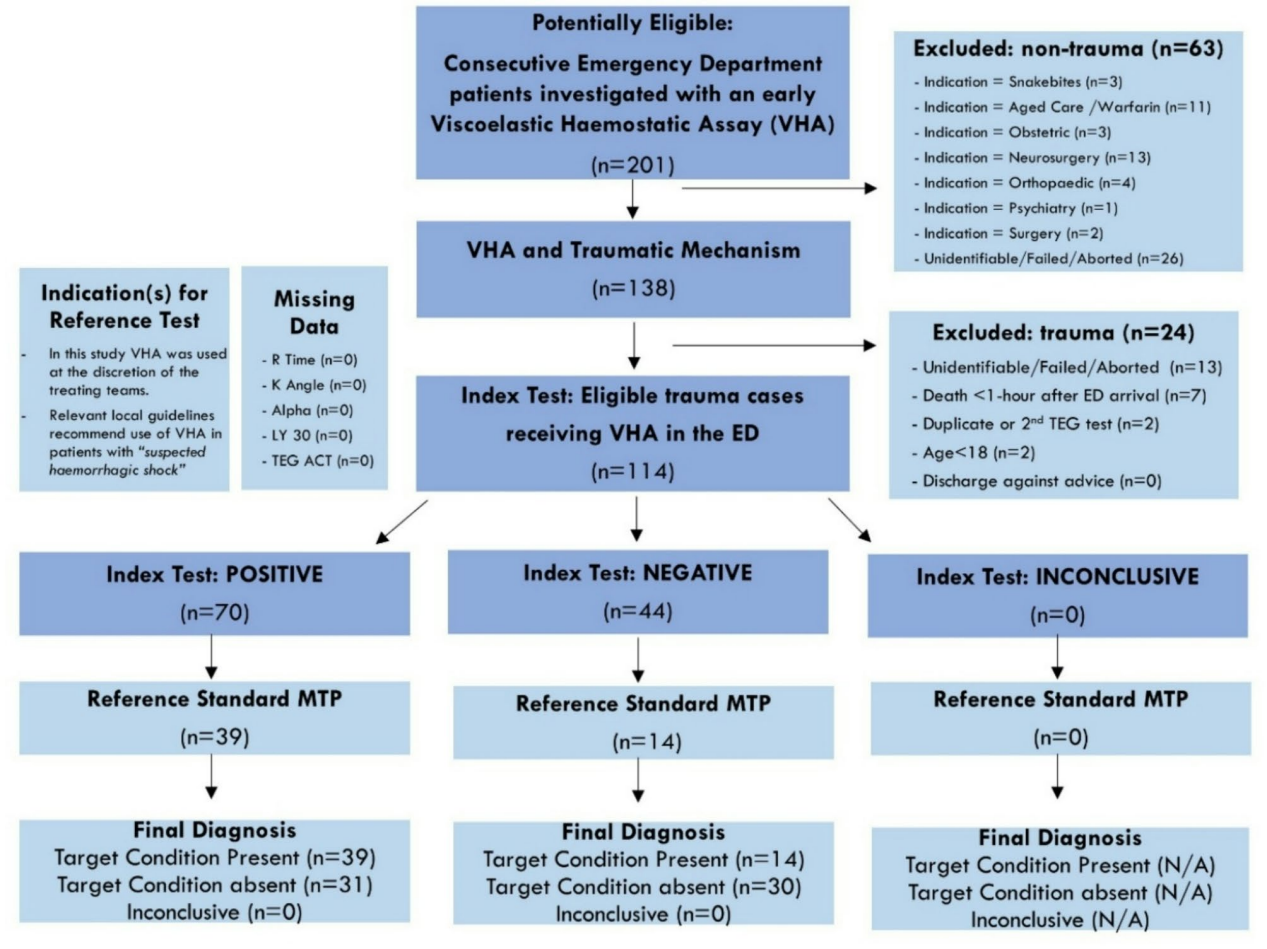
STAndards for the Reporting of Diagnostic accuracy studies (STARD) diagram

VHA was introduced at the study site (Westmead Hospital) in November 2017. Within the 3-year study window (1st January 2018 to 31st January 2021), local trauma guidelines ‘recommended’ using the VHA device in the event of major trauma. The eligibility criteria for the study are outlined in the STARD diagram (Fig. 1).

The inclusion criteria were: (i) Adult patients with traumatic injury in the Emergency Department; (ii) TEG test initiated within 1-hour of ED triage. The exclusion criteria were: (i) non trauma presentation; (ii) TEG test ‘unidentifiable’ or ‘unmatched’ to trauma a case; (iii) TEG test aborted < 30 min (iv) Age < 18; (v) discharge against medical advice; (vi) death within 1-hour of ED triage.

Regarding the Massive Transfusion (MT) definitions, an outcome of “*MT received*” was defined by a definition: (i) a total transfusion of ≥ 10 units PRBC within 24-hours *OR* (ii) any transfusion of Packed Red Blood Cells (PRBC) ≥ 6 units within 4-hours of admission. MT as the primary endpoint choice should be noted. As a result, while all patients were “major trauma” activations with ISS > 12, not all patients who were included had MHP activated (i.e., 74.8% did); and not all patients received transfusion (i.e., 8.8% did not).

### VHA index test

VHA tests measure viscoelastic properties of blood under low-shear stress [15]. The device used was a *TEG6s™* (*Haemonetics*,* Braintree*,* MA*,* USA*). We engaged with peer-hospitals that are regular research collaborators but found these collaborators were adopting ROTEM rather than TEG which precluded expanding this study.

The VHA machine was available as a point-of-care (POC) test (i.e., in the trauma bay). Lloyd-Donald and co-authors suggested that the TEG6s is a broadly generalisable test but there are no specific validation studies in the trauma population [20]. However, during the study period according to the local policy VHA was recommended for “*patients at risk of requiring large volume transfusion*”.

Device training was provided staff (*n* = 72) prior to implementation and ad-hoc during the study. As per the manufacturer specifications the machine used a citrated blood sample deposited into a cartridge-based channel. Cartridges use dried reagents in four tests, each with calcium chloride (to reverse citrate) and additional specific test reagents designed to assess various parts of coagulation: (i) citrated kaolin (CK); (ii) kaolin / tissue factor (RapidTEG); (iii) kaolin / abciximab (*citrated functional fibrinogen (CFF*)). The VHA device results and reference ranges included:

(i) R-time - Reference Ranges - CK 4–8 (mins); RapidTEG 0.3–0.8 (mins).
(ii) K-time - Reference Ranges - CK 1–4 (mins); RapidTEG 0.5–2.3 (mins).
(iii) α-angle - Reference Ranges - CK 47–74 (degrees); RapidTEG 64–80 (degrees).
(iv) Maximum amplitude (MA) - Reference Ranges - CK 55–73 (mm); RapidTEG 52–71 (mm).
(v) Lysis% at 30 min (LY30) - Reference Ranges - CK 0–8 (%); RapidTEG 0–5 (%).
(vi) CFF - Reference Range - 11–24 (mm).
(vii) TEG6s Activated Clotting Time (ACT) - Reference Range 82–152 (seconds).

### Outcomes measures

The primary outcome measure was absence (or presence) of a confirmed MHP within 24-hours of ED arrival recorded as a binary outcome. Further a priori data points collected included (i) demographics (age, mechanism of injury, gender, ISS, vital signs); (ii) trauma investigations (documented examination, blood testing including INR (with a cut-off of ≥ 1.5 to estimate the presence or absence of TIC), radiology and sonography); (iii) transfusion administered within < 24 h (iv) hospital length of stay and mortality; (iv) calculated clinical decision triage scores (with the accepted cut-points for predicting MHP which are listed in Table 1): [21–25]

Reference data for all admitted trauma cases presenting during the same time-window were available. This data is part of an established trauma registry that only captures patients with ISS ≥ 12. We purposefully included a comparison to registry patients in Table 2. The rationale was inclusion for ‘reference only’. This adds to context and transparency about the level of injury in the cohort and thereby the generalisability of the findings.

**Table 1:** Study population characteristics and reference group

	Test Statistic	Enrolled patients: Eligible Study Population (VHA Performed) (*n* = 114)	** Reference data for trauma admissions: (All Major Trauma Calls in study window with ISS > 12) (*n* = 1753)	*p*-value
Demographics	Age (mean, SD)	45.3 (19.5)	53.7 (22.9)	< 0.0001
	Male (n/%)	83 (72.8%)	1293 (73.8%)	N/A
	Female (n/%)	31 (27.2%)	460 (26.2%)	
	Non-Binary/Other (n/%)	0 (0%)	0 (0%)	
Transfusion	PRBC at 24 h (mean, SD)	4.3 units (SD 5.0)	0.73 units (SD 3.0)	< 0.0001
> 1unit PRBC received	ED or pre-hospital (n/%)	104 (91.2%)	N/A	N/A
Vitals	Systolic BP (mmHg) (mean, SD)	114.3 (27.1)	131.0 (28.1)	< 0.0001
	Diastolic BP (mmHg) (mean, SD)	73.5 (22)	77.2 (16.9)	0.029
	***Heart rate (bpm) (mean, SD)	101.8 (24.4)	85.4 (22.0)	< 0.0001
	Respiratory rate (mean, SD)	18.9 (5.6)	18.4 (4.5)	0.26
Mortality	In-hospital mortality (n/%)	8 (7.0%)	109 (6.2%)	0.39
Injury	*ISS (mean, SD)	19.7 (11.3)	17.4 (8.2)	0.0067
Mechanism	Blunt (n/%)	91 (79.8%)	1644 (93.8%)	N/A
	Penetrating (n/%)	23 (20.2%)	109 (6.2%)	
Laboratory Results	Haemoglobin (mean, SD)	130.1 (22.5)	N/A	N/A
	pH (mean, SD)	7.31 (0.1)	N/A	N/A
	Base excess (mean, SD)	-3.4 (5.0)	-3.4 (4.9)	0.96
	Lactate (mmol/L) (mean, SD)	3.5 (3.9)	3.3 (4.2)	0.70
	PT (seconds) (mean, SD)	15.4 (3.3)	N/A	N/A
	APTT (seconds) (mean, SD)	31.6 (10.8)	N/A	N/A
	International Normalised Ratio (INR) ≥1.2 (n%)[26]	43 (37.8%)	257 (27.7%)	0.023
	International Normalised Ratio (INR) ≥1.5 (n%)	11 (9.6%)	76 (6.4%)	0.19
	Fibrinogen	2.5 (0.9)	N/A	N/A
	Platelets (mean, SD)	228 (149.9)	N/A	N/A

Table Footnote - **Column included for reference only; ***beats per minute (bpm); * injury severity score (ISS)

**Table 2:** Test performance for prediction of transfusion within 24-hours

VHA Test Accuracy Summary							Was a massive transfusion received?		Total (%)	
							*Yes (%)*	*No (%)*		
**Primary Outcome**: ANY abnormal VHA Component* *AUROC 0.633* *[0.531–0.736]*	Sensitivity 73.6% (59.7-84.7%)	Specificity 49.2% (36.1-62.3%)	PPV 55.7% (48.4-62.2%)	NPV 68.2% (56.1-78.2%)		Abnormal	39 (34.2%)	31 (27.2%)		70 (61.4%)
						Normal	14 (12.2%)	30 (26.3%)		44 (38.6%)
TEG6s Activated Clotting Time (ACT)	Sensitivity 18.9% (9.4-32.0%)	Specificity 80.3% (68.2-89.4%)	PPV 45.5% (28.2-63.9%)	NPV 53.3% (48.8-57.7%)		Abnormal	10 (8.7%)	12 (10.5%)		22 (19.3%)
						Normal	43 (37.7%)	49 (43.0%)		92 (80.7%)
R (min) CK (Standard TEG)	Sensitivity 32.1% (19.9-46.3%)	Specificity 77.1% (64.5-86.9%)	PPV 54.8% (39.9-69.0%)	NPV 56.6% (50.9-62.2%)		Abnormal	17 (14.9%)	14 (12.2%)		31 (27.2%)
						Normal	36 (31.5%)	47 (41.2%)		83 (72.8%)
K (min) CK (Standard TEG)	Sensitivity 26.4% (15.3-40.3%)	Specificity 88.5% (77.8-95.3%)	PPV 66.7% (46.6-82.1%)	NPV 58.1% (53.5-62.5%)		Abnormal	14 (12.3%)	7 (6.1%)		21 (18.4%)
						Normal	39 (34.2%)	54 (47.4%)		93 (81.6%)
a-angle (deg) CK (Standard TEG)	Sensitivity 18.9% (9.44-32.0%)	Specificity 83.6% (71.9-91.9%)	PPV 50.0% (31.1-68.9%)	NPV 54.3% (50.0-58.5%)		Abnormal	10 (8.8%)	10 (8.8%)		20 (17.5%)
						Normal	43 (37.7%)	51 (44.7%)		94 (82.4%)
MA (mm) CK (Standard TEG)	Sensitivity 28.3% (16.8-42.4%)	Specificity 85.3% (73.8-93.0%)	PPV 62.5% (44.3-77.7%)	NPV 57.8% (52.9-62.5%)		Abnormal	15 (13.1%)	9 (7.9%)		24 (21.0%)
						Normal	38 (33.33%)	52 (45.6%)		90 (78.9%)
LY30 (%) CK (Standard TEG)	Sensitivity 3.8% (0.5-13.0%)	Specificity 96.7% (88.7-99.6%)	PPV 50.0% (12.7-87.3%)	NPV 53.6% (51.9-55.4%)		Abnormal	2 (1.8%)	2 (1.8%)		4 (3.5%)
						Normal	51 (44.7%)	59 (51.8%)		110 (96.5%)
ANY abnormal “Rapid” TEG *AUROC 0.582* *[0.477–0.688]*	Sensitivity 43.4% (29.8-57.7%)	Specificity 73.8% (60.9-84.2%)	PPV 59.0% (46.0-70.8%)	NPV 60.0% (53.2-68.7%)		Abnormal	23 (20.2%)	16 (14.0%)		39 (34.2%)
						Normal	30 (40%)	45 (60%)		75 (65.8%)

Table Footnote - Positive Predictive Value (PPV); Negative Predictive Value (NPV); Confidence Interval (CI) [* VHA components include the following: R-CK, K-CK, Alpha Angle CK, MA- CK, LY30 CK, CFF-MA and any rapid TEG]

### Data analysis and statistical plan

The estimated sample size was a pragmatic consideration. An initial enrolment target of two hundred recommended by the consulting statistician (KB). All eligible cases within the predefined study period were included. Data were analysed using IBM SPSS (V24) by a consulting statistician. Mean and standard deviation (SD) were used to summarise continuous variables. Frequencies and percentages were used for categorical variables. Evaluation of statistical differences between the general trauma population and study population were evaluated using Student’s t-test or chi-square tests as appropriate. A 2-tailed *p* < 0.05 was considered significant and the mean differences are reported together with 95% confidence intervals (CIs) as appropriate.

VHA data from the device was downloaded via the proprietary TEG-manager™ system and we used manufacturers’ cut-points that could suggest coagulopathy (i.e., hypocoagulability) to define abnormal versus normal results and therefore create a binary outcome for the index test. Area under the curve (AUROC) was calculated for the primary outcomes and for each clinical decision score. Sensitivity, specificity and predictive values were derived for each component of the VHA using the STARD approach (Fig. 1).

## Results

Within the study window, 9,037 trauma presentations were reported and 286 MHP activations. There were 201 VHA tests collected in ED with 154 classified as trauma. After inclusion criteria were applied 114 patients were eligible.

91.2% received at least one unit of packed red cells. No cases with index test results were lost to the 24-hour follow-up. Patients in the index test cohort when compared with the local trauma registry population had significantly higher ISS, were younger and received more blood components than the general trauma population with ISS ≥ 12 reflecting the population was at risk of haemorrhage and requirement for MHP.

Table 3 outlines the results for primary outcome and test accuracies. *VHA-normal* versus *VHA-abnormal* groups are directly compared. Results for the accuracy of the index test are also presented in two-by-two tables. Among patients investigated with VHA the mean results for the standard cartridge test (Citrated Kaolin) were as follows: Reaction Time (R) − 5.7 min (SD 1.9) (normal range 4.6–9.1); Kinetics (K) − 1.8 min (SD 1.3) (normal range 0.8–2.1); Alpha Angle (α) − 68.7 degrees (SD 7.6) (normal range 63–78); MA = Maximum Amplitude (MA) − 56.7 mm (SD 9.0) (normal range 52–69); Lysis at 30 min (LY%) − 0.44% (SD 0.8) (Normal Range 0-2.6).

In terms of other specific calculated test accuracies CFF MA (mm) Sensitivity was 34.0% (95% CI 21.5%-48.3%) and Specificity 86.9% (95% CI 75.8%-94.2%) for predicting MHP. The calculated sensitivity for detecting TIC in this population was 75% (95% CI 42.8%-94.5%) using a cut point INR ≥ 1.5. Specificity for TIC was 41.2% (95% CI 31.5–51.3) [26]. 

**Fig. 2:**
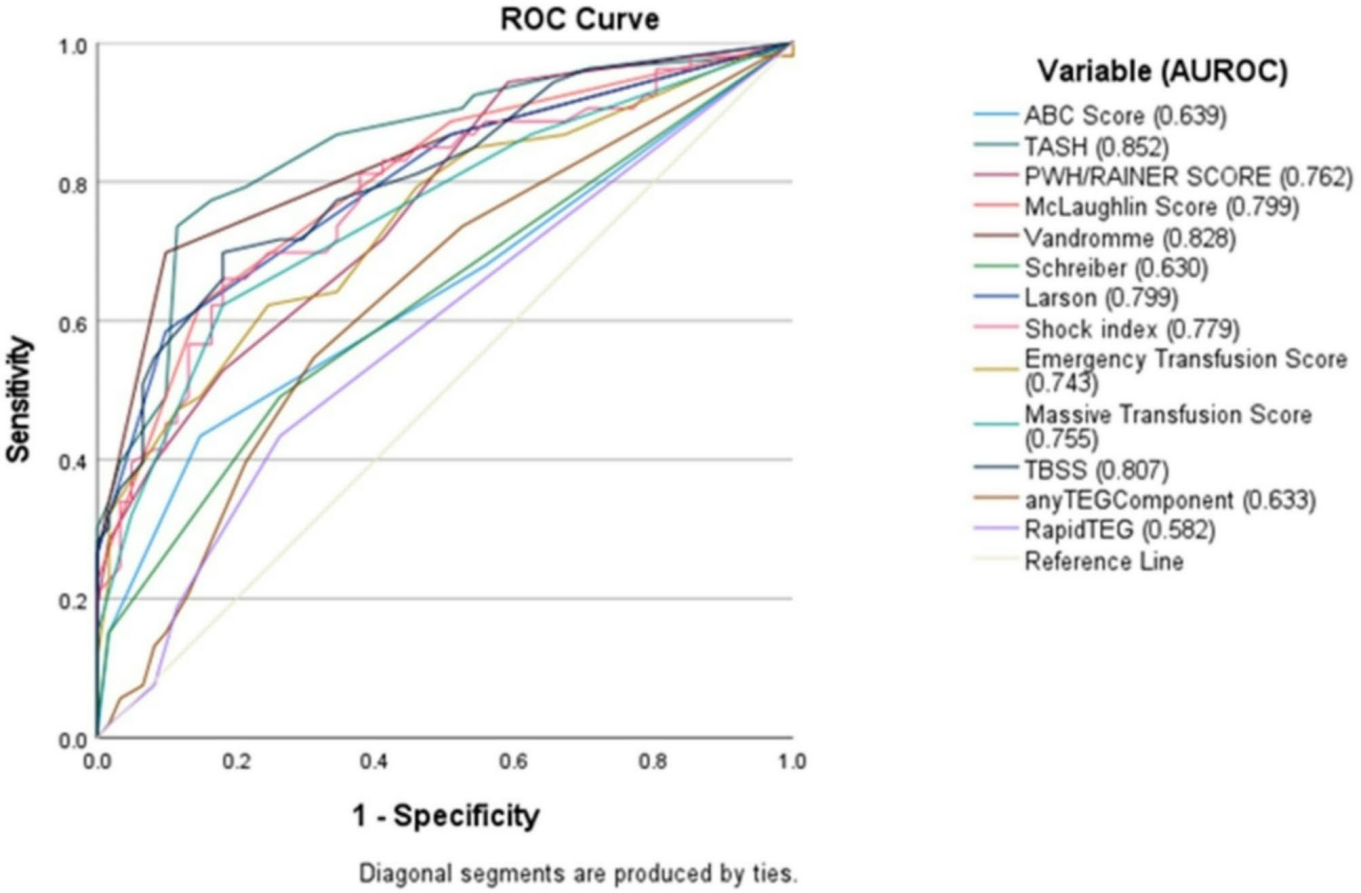
Receiver operating curves for validated transfusion prediction score

Figure 2 illustrates the secondary outcome of adding a VHA result to existing decision scores for MHP prediction. The addition of VHA to existing clinical decision triage scores with standard cut points (Table 1) was noted to be of marginally additive value in predicting administration of MHP within 24-hours.

**Table 3:** Viscoelastic haemostatic assay test combined with validated decision scores (*n* = 114)

**Validated clinical decision triage scores**	**Score Accuracies for predicting massive transfusion at 24-hours (calculated from MHP clinical triage scores alone)**					Screening Estimated Accuracies (calculated from - any “abnormal” VHA test result *OR* abnormal MHP clinical decision triage score result)				Estimated Accuracy (combining theoretical test that includes *BOTH* VHA test result *and* MHP clinical decision triage score result = “*abnormal*”)			
	***Sensitivity***	***Specificity***	***AUC [95% CI)***	***PPV***	***NPV***	***Sensitivity***	***Specificity***	***PPV***	***NPV***	***Sensitivity***	***Specificity***	***PPV***	***NPV***
**ABC**	43.4%	85.2%	0.639 [0.535, 0.743]	71.8%	63.4%	86.8%	40.1%	56.1%	78.1%	30.2%	93.4%	80.0%	60.6%
**TASH**	77.3%	83.6%	0.852 [0.780, 0.924]	80.5%	80.9%	96.2%	45.9%	60.7%	93.3%	54.7%	86.9%	78.4%	68.8%
**Shock Index (Cut Point 0.8)**	84.9%	54.1%	0.779 [0.693, 0.866]	61.6%	80.5%	94.3%	31.2%	54.4%	86.4%	64.2%	72.1%	66.7%	69.8%
**Shock Index (Cut Point 1)**	62.3%	83.6%	0.779 [0.693, 0.866]	76.7%	71.8%	92.5%	41.0%	57.7%	86.2%	43.4%	90.2%	79.3%	64.7%
**PWH/R**	52.8%	81.9%	0.762 [0.676, 0.848]	71.8%	66.7%	88.7%	41.0%	56.6%	80.7%	37.7%	90.2%	76.9%	62.5%
**McLaughlin**	N/A	100%	0.799 [0.717, 0.881]	N/A	53.5%	73.6%	49.2%	55.7%	68.2%	N/A	100.0%	N/A	53.5%
**Vandromme**	69.8%	90.1%	0.828 [0.748, 0.907]	86.1%	77.5%	96.2%	26.2%	53.1%	88.9%	64.2%	72.1%	66.7%	69.8%
**Schreiber**	49.0%	73.7%	0.630 [0.527, 0.734]	61.9%	62.5%	92.5%	32.8%	54.4%	83.3%	30.2%	90.2%	72.7%	59.8%
**Larson**	58.4%	90.1%	0.799 [0.716, 0.882]	83.8%	71.4%	92.5%	44.3%	59.0%	87.1%	30.2%	90.2%	72.7%	59.8%
**ETS**	64.2%	65.6%	0.743 [0.652–0.835]	61.8%	67.8%	96.2%	23.0%	52.0%	87.5%	56.6%	80.3%	71.4%	68.0%
**MTS (excluding sonography result)**	32.1%	95.1%	0.755 [0.655–0.846]	85.0%	61.7%	86.8%	41.0%	56.1%	78.1%	43.4%	90.2%	79.3%	64.7%
**TBSS**	39.6%	93.4%	0.807 [0.727–0.887]	84.0%	64.0%	86.8%	41.0%	56.1%	78.1%	32.1%	93.4%	81.0%	61.3%

Table Footnote - Positive Predictive Value (PPV); Negative Predictive Value (NPV); Confidence Interval (CI)

## Discussion

VHA results return faster and are strongly associated with clinical outcomes of interest to clinicians caring for severely injured trauma patients [27]. In this exploratory study the use of a new point of care VHA device located in the ED was assessed. We included data from the first three years of use with similar methods to two parallel studies [28, 29]. The use of VHA in operating room settings has been well described [27] but to date there is limited reporting of use in a pre-hospital or ED setting [30–32]. One potential advantage of use in the ED previously recognised in the literature is the potential for early recognition of coagulopathy compared to conventional laboratory testing [33]. 

In this study we observed that VHA displayed moderate specificity for predicting MHP as a “*rule in*” test (Table 3). For example, when activated clotting time (a rapid part of the VHA test) was abnormal the specificity for predicting MHP was 80.3% (95%CI 68.2-89.4%). VHA individual parameters performed modestly, ranging between 77% and 97%.

Citrated functional fibrinogen (CFF) had the best performance with an 86.9% specificity for predicting MHP. These findings are not surprising. The CFF is sometimes considered a proxy measure for fibrinogen levels (currently recruiting Feisty 2 study). CFF having the best predictive value in this study is consistent with the wider literature which suggests this component is depleted in patients receiving MHPs [28]. 

While VHA may some very limited utility as a “rule out” test MHP initiation ideally should be over-called in the early stages of care [8]. We observed a standalone sensitivity of 73.6% (95% CI 59.7%-84.7%) for predicting MHP. This is consistent with similar studies [8]. This level of performance does not justify VHA as a either a rule out or screening test [34]. 

Given INR is recognised as a predictor of MHP, as an alternative approach to predicting MHP we combined the VHA results with validated clinical decision scores (Table 1). Using this strategy we observed (as expected) a higher level of sensitivity at the loss of specificity. While further study in larger trials would be required for validation, these results suggest the VHA could be used as a “rule out” test of moderate value when accounted for in the clinical context or combined into a new score. This conclusion is in keeping with work by Hagemo et al. who state that VHA was a *valid marker for TIC and predictor for MT*, reporting a 77% sensitivity for predicting MHP [8]. Future use of VHA in the ED could assist clinicians in predicting the risk of requiring MHP albeit with the caveats listed. To avoid wastage of blood components following an initial “over calling” strategy, de-escalating of the MHP could be aided by assessing the VHA, patient condition and laboratory values trending towards improvement. For a treating trauma team, the take-home message would be that a normal TEG confers a lower risk of needing an MHP but cannot be used as a standalone test. Likewise, any significant abnormality especially with the CFF should raise concerns about a need for escalation to an MHP if this has not already occurred.

The study was hindered by slow recruitment and has a small cohort when compared to retrospective European studies. One of these studies was from France. These authors concluded VHA was associated with more “*patients alive and free of MHP delivery at 24 hours”* and a *“reduction of blood component use/costs*” [29]. Our cohort size undermines our ability to make similarly robust conclusions. The lack of volume we observed is partially predictable given our Australian setting where trauma systems see a relatively diluted volume of severe trauma cases [35–37]. Despite these limitations, we note that the accuracies observed is comparable with other studies in ED practice that are generally accepted as valid for everyday practice [38–42]. The rates of coagulopathy (TIC) observed in this study (INR ≥ 1.5 cut point) were also similar to comparable prior studies [43–45]. 

A decision to activate MHPs is based on various factors including vital signs, mechanism of injury, injury severity and examination findings [23]. It is tempting to make such decisions based on gestalt rather than relying on clinical scores, teamwork or negotiation [46, 47]. As noted a secondary aim was to consider whether the addition of VHA results to decision tools (Table 1) would increase diagnostic value. The observed effect of adding VHA to the validated MHP decision scores increased overall sensitivity which may suggest a converse role for VHA in “turning off the tap” In other words, in trauma patients with reassuring symptoms, signs, investigations, normal VHA and low MHP prediction score we may be able to discuss cancelling the MHP within the team. Further work would be required to confirm the broad validity of these concepts.

Turning to cost, we note that recent studies in both trauma and cardiac surgery suggested that VHA-guided transfusion reduced blood component use and consequent transfusion costs significantly [16, 48]. If this were also true in the ED setting this would be of significant interest to trauma teams managing patients on arrival to hospital. VHA which costs approximately $150(USD) per patient but MHPs are also expensive [28, 49]. While this study did not directly measure expenditure we note that predictive value for MHP reported could contribute to rationalisation of MHPs and thereby cost [29, 30]. The jury is still out as to whether VHA use is cost effective with four before-after observational studies giving mixed results [28, 32]. Recent work by Cochrane et al. demonstrated VHA-driven algorithms were associated with reduced mortality and reduced product wastage justifying further studies on the question of cost-effectiveness [28, 50].

In terms of additional limitations, we would like to acknowledge that 8% of the cohort did not receive blood components. By comparison, most similar studies have included only patients that received transfusion [29]. The sensitivities observed relate to predicting MHP within 24-hours which only inferentially informs us about the utility of the test (Fig. 1). Therefore, we recommend when faced with an undifferentiated trauma patient the clinician must adopt a low threshold for MHP initiation. We acknowledge there are limitations in the use of both potential primary endpoints: (i) delivery of a massive transfusions (MT) or (ii) activation of a major haemorrhage protocols (MHP). Delivery of MT is limited by effect and survival bias, while measuring activation of MHPs are limited by selection bias. We used MT for the primary outcome as similar studies have used this endpoint. A further limitation of this work is the observational design which could lead to selection biases. Exclusion of cases with pre-hospital or early death (< 1 h) could create a vanguard selection (mortality bias) issue that could be exaggerated due to a small sample. As Cassar et al. note in their editorial accompanying the ITACTIC study randomised trials are needed *“[sic] before broad implementation of a new strategy*” [5, 51]. We also note that treating doctors may or may not have used the VHA in their plans for individual patients. The study was limited by recruitment with 201 uses of the device during the study window. For readers outside Australia, there is a risk of extrapolation to other settings due to the single-site and geographical location.

In conclusion, the results presented are not sufficient to recommend that VHAs are relied on as a standalone test for MHP prediction. Further studies should examine if VHA test parameters can be added to (or replace INR) in existing clinical scores used to make decisions about transfusion in trauma patients such as the Vandromme score.

## Electronic supplementary material

Below is the link to the electronic supplementary material.

Supplementary Material 1

## Data Availability

No datasets were generated or analysed during the current study.
